# Expanding the phenotype in Pitt-Hopkins syndrome; description of new oral finding and dental management considerations

**DOI:** 10.1186/s12903-024-04296-5

**Published:** 2024-05-22

**Authors:** Yazan Hassona, Dua’a Alqaisi, Asma Alkilani, Iyas AbuHijleh

**Affiliations:** 1 Faculty of Dentistry, Centre for Oral Diseases Studies, Al-Ahliyya Amman University, As-Salt, Jordan; 2 School of Dentistry, The University of Jordan, As-Salt, Jordan; 3 School of Medicine, The University of Jordan, As-Salt, Jordan

**Keywords:** Oral, Health, Pitt-Hopkin, Syndrome, TCF4

## Abstract

**Background:**

Pitt-Hopkins syndrome (PTHS) is a rare neurodevelopmental disorder with physical, cognitive, and behavioral characteristics that is caused by heterozygous mutations in the *TCF4* gene. Patients with PTHS might present a unique challenge for oral healthcare professionals because of the associated comorbidities.

**Case report:**

Here we describe a new case of PTHS in a 13-year-old girl with particular emphasis on oro-dental findings and oral healthcare management. Observed oro-dental findings in our case included shallow palate, absence of lingual frenum, gingival enlargement, thick lips and relative microdontia. The patient was unable to tolerate dental care under local anesthesia. Therefore, comprehensive dental treatment was performed under general anesthesia after a careful pre-anesthetic cardio-respiratory, neurological, and hematological evaluation. The patient was closely monitored intra-operatively for breathing rhythm, O2 saturation, and signs of respiratory distress. The patient was observed for 24 h post-op for respiratory distress and was discharged then uneventfully.

**Conclusion:**

Dental treatment under general anesthesia in these patients might be complicated by the abnormal breathing rhythm, and close monitoring and follow up for signs of respiratory distress after general anesthesia is necessary. Recognition of oral and dental findings might help to expand the phenotype and better characterize rare syndromes.

## Introduction

Pitt-Hopkins syndrome (PTHS) is a rare autosomal dominant neurodevelopmental disorder that was described for the first time in 1978. The Australian pediatricians David Pitt and Ian Hopkins, during a survey of 782 individuals with learning disability, identified two unrelated children with characteristic facial appearance, intellectual disability, abnormal breathing rhythm and abnormal electroencephalograms [1]. However, the syndrome was only recognized as a distinct entity in 2007, when Zweier et al., using molecular karyotyping with SNP arrays, identified de novo mutations in the *TCF4* gene as the cause of the syndrome [2–4]. *TCF4* is a protein-coding gene that is located at the long arm of chromosome 18. It encodes the transcription factor 4 (TCF-4) which is expressed in the embryonic central nervous system and plays important roles in neural differentiation and development [5–7]. 

The phenotype of Pitt Hopkins Syndrome (PTHS) is characterized by a distinctive set of features encompassing neurodevelopmental, respiratory, and facial aspects. Individuals with PTHS typically present with moderate to severe intellectual disability, generalized developmental delay, hypotonia, episodes of hyperventilation followed by apnea and cyanosis and episodes of seizures and constipation [3, 8]. Furthermore, they may exhibit distinctive stereotypic rolling head movements and hand movements such as clapping, washing, wringing, and flapping with a happy disposition. Occasional episodes of anxiety, aggression, and sleep disturbances are also common [3, 8, 9]. The diagnosis of PTHS might be challenging because of the relatively non-specific features, especially during infancy and early childhood. Clinical diagnostic criteria that includes both cardinal and supportive features have been proposed (Table 1).

Patients with PTHS exhibit characteristic facial features including microcephaly, bitemporal narrowing, cup-shaped and low set ears with a thick helix, myopia, astigmatism, strabismus, and thin midline eyebrows. In addition to, the nose is often large with a broad bridge and flared nostrils, and the mouth is wide with fleshy-full lips and an M-shaped Cupid’s bow [9–13]. 

Oro-dental features in PTHS are only sparsely reported and include shallow and broad palate with widely spaced teeth, and mandibular prognathism [11, 12]. Oral health care management in affected patients might be complicated by learning disability, hypotonia, seizures, and abnormal chewing and eating habits. In the present report, we describe a new case of PTHS with emphasis on oro-dental findings, and describe the challenges in providing oral healthcare for individuals with PTHS.

## Case report

### History of present illness

A 13-year-old girl with severe learning disability presented to the Special Care Dentistry clinic with the complaints of “toothache, sialorrhea, bad breath, and decreased oral intake”. Her mother reported that she became agitated, anxious, refused eating or drinking, and clenched at her teeth for most of the day during the last week. The family tried over the counter pain killers but there was no improvement.

### Medical and social history

The girl was born at full term normal delivery to non-consanguineous parents, and her medical history was unremarkable for systemic diseases, drug allergy, or use of medications. The family reported that the girl never spoke nor walked since she was born. Furthermore, the mother noticed an abnormal breathing pattern since birth, especially at night, characterized by periods of hyperventilation followed by shorter periods of apnea. Whole- exome sequencing performed at the age of five concluded the presence of heterozygous variant (a de novo stop gain mutation) in the gene *TCF4* (NM_001243226.2): c.[1459 C > T].[(Arg487*)]; confirming the genetic diagnosis of PTHS. The girl never went to school because of learning disability, but the family reported that they send her occasionally to a rehabilitation center for people with intellectual disability. Th parents denied any history of epilepsy or episodes of seizure.

### Physical examination

Physical examination carried out when the patient was 13 years old revealed global developmental and growth delay (height = 128 cm [5 percentile]; weight 27 kg [10 percentile]).

**Fig. 1:**
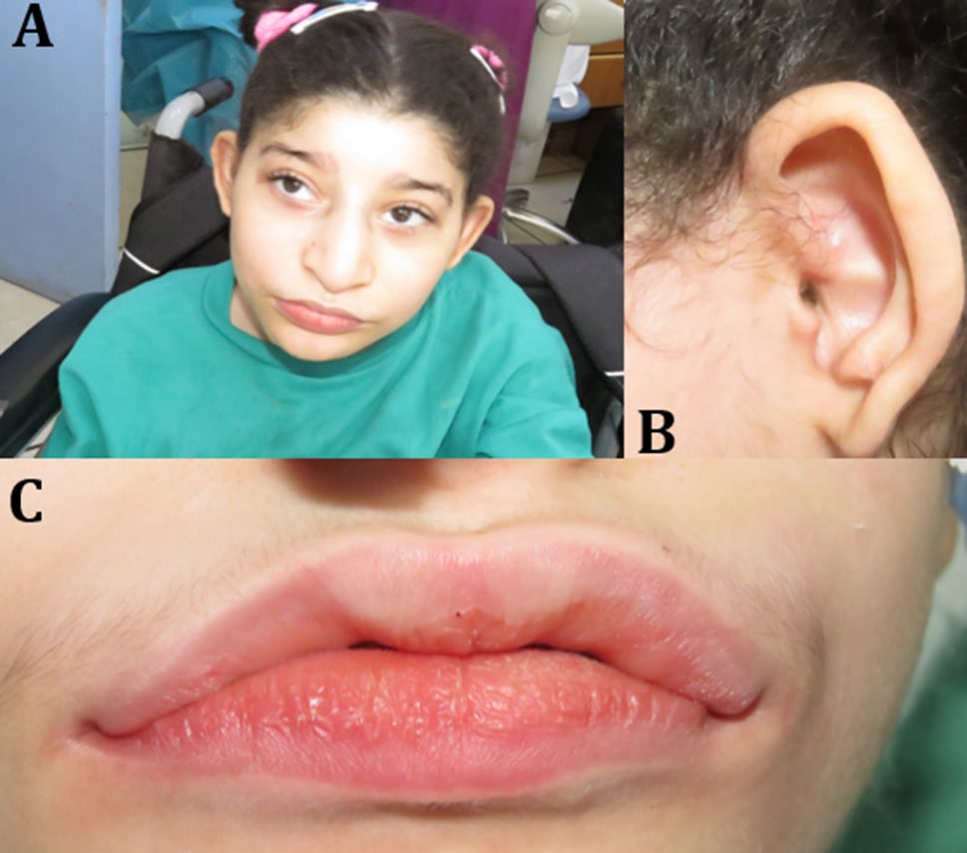
Clinical features of PTHS, (**A**) Typical facial appearance of PTHS, (**B**) Cup-shaped ear, (**C**) Thick, fleshy lips with M-shaped cupid bow

**Fig. 2:**
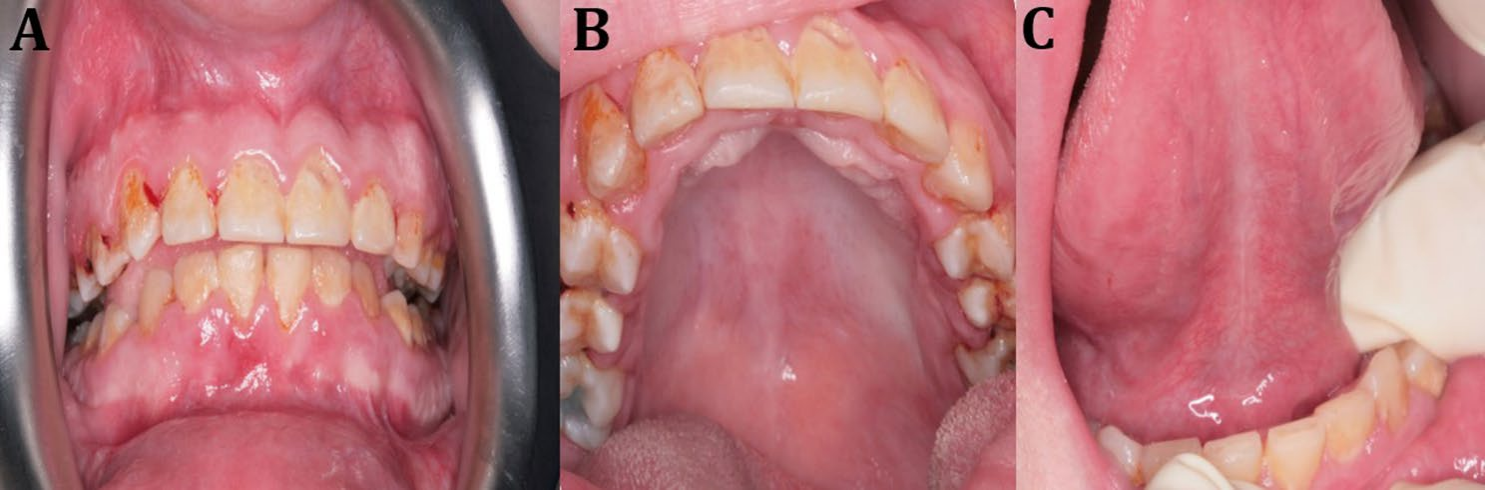
Oral findings in PTHS, (**A**) Generalized gingival inflammation and enlargement, (**B**) Broad U-shaped palate, (**C**) Absence of lingual frenum

Head and neck examination revealed brachycephaly, strabismus, prominent and long eyelashes, up-slanted palpebral fissures, cup-like ears, and wide nasal bridge. Her mouth was wide, and her lips and cheeks were full and fleshy (Fig. 1). Intra-oral examination was performed while the patient was sitting in her wheelchair, and showed generalized attrition, microdontia, multiple carious lesions on upper and lower teeth with badly destructed lower left first molar. Soft tissue examination revealed generalized gingival enlargement, shallow and U-shaped wide palatal vault, absence of lingual frenum (Fig. 2). In order to exclude the possibility that the lingual frenum is hidden or being submucosal, the tongue was elevated and placed posteriorly, nevertheless there was no evidence of the presence of lingual frenum. Salivary flow was within normal range and the saliva was of normal consistency however the patient was presented with clinical evidence of sialorrhea due to muscular hypotonia. Her fingers and toes were thin, tapered, and slightly clubbed, and she exhibited hand flapping and clapping behavior. The case evolved with hypotonia and inability to respond to verbal or non-verbal communication.

### Oral healthcare management

Comprehensive dental treatment was planned under general anesthesia because she was unable to tolerate dental treatment under local anesthesia. Pre-anesthetic assessment included cardio-respiratory, neurological, and hematological evaluation. All clinical assessment and laboratory tests (i.e. complete blood count (CBC), liver function and kidney function tests (LFT/KFT), and arterial blood gases (ABGs)) were within normal. Naso-endotracheal intubation was performed smoothly in the semi supine position because of difficulty in lying down, and dental treatment, including extraction of an unrestorable lower left first molar, restorative treatment of dental caries, and gingival prophylaxis, was performed in a single session. The patient was agitated slightly upon extubation with normal O2 saturation (97%) and normal deep breaths and respiratory rate. The patient was observed for 24 h post-op for any signs of respiratory distress and was discharged then uneventfully.

The mother explained that she doesn’t know how to brush her teeth and was afraid of causing her any accidental injury while brushing. Furthermore, she reported that her diet was based mainly on soft food because of the difficulty in swallowing and the long chewing time. Tailored oral hygiene instructions and dietary advice were given to parents. The patient was placed on a three-monthly oral hygiene promotion schedule.

## Discussion

PTHS is a neurodevelopmental syndrome with physical, cognitive, and behavioral characteristics. Worldwide there is less than 500 cases of PTHS; however, the syndrome might be underdiagnosed or misdiagnosed because of the heterogenous clinical presentation, lack of universally accepted diagnostic criteria, and the phenotypic similarity with other neurodevelopmental syndromes such as Angelman syndrome and Rett syndrome [8, 11, 14]. In 2019, the 1st international consensus statement regarding diagnosis and management of PTHS was released and clinical diagnostic criteria, based on a thorough review of 100 patients with confirmed molecular diagnosis, was proposed (Table 1) [15]. Nevertheless, the proposed diagnostic criteria didn’t include any mention of specific oral or dental findings as a diagnostic component in PTHS; probably because of lack of reports describing dental and oral findings in these patients. Reporting oral and dental findings in patients with PTHS are therefore important because this might help in refining the current diagnostic criteria and differentiating PTHS from other similar neurodevelopmental disorders such as Angelman and Rett syndromes.

**Table 1:** Clinical diagnostic criteria for PTHS

Cardinal features	Supportive features
**1. Face** (at least three of seven) a. Narrow forehead b. Thin lateral eyebrows c. Wide nasal bridge/ridge/tip d. Flared nasal alae e. Full cheeks/ prominent midface f. Wide mouth/full lips/cupid bow upper lip g. Thickened/overfolded helices *4 points* **2. Severe intellectual disability with absent or limited speech** (< 5 words) *2 points* **3. Breathing regulation anomalies** (intermittent hyperventilation and/or apnea) *2 points*	**1. Myopia** **2. Constipation** **3. Hand** (slender fingers and/or abnormal palmar creases) **4. Unstable gait** *each 1 point*
- **Clinical diagnosis of Pitt-Hopkins syndrome** (Score ≥ 9) Molecular confirmation indicated. - **Possible clinical diagnosis of Pitt-Hopkins syndrome (**Score of 6–8) Presence of facial characteristics + additional criteria, either cardinal or supportive, totaling a Score of 6–8. This score warrants TCF4 molecular analysis. - **Insufficient clues for the presence of Pitt-Hopkins syndrome (**Score < 6) No further studies specifically for PTHS indicated. Further studies for other etiologies indicated.	

In addition to genetic confirmation, our patient demonstrated the characteristic facial features and exhibited severe intellectual disability, breathing regulation abnormalities, and slender fingers; therefore, meeting the diagnostic criteria of the international consensus statement of PTHS [15]. 

Oral and dental examination in our patient revealed relative microdontia, multiple caries, generalized gingival enlargement, and shallow U-shaped palate. It might be difficult to compare our oro-dental findings with previously published literature because of the lack of adequate description and specific reports about dental findings in PTHS. Nevertheless, the reported oro-dental findings in the present case are, in the main, non-specific and similar to reported findings in other neurodevelopmental disorders such as Angelman syndrome, Rett syndrome, Dandy-Walker, Shashi-Pena syndrome and autism spectrum disorder [8, 15, 16].

The presence of dental caries in our patient can be explained by the lack of adequate hygiene practices, the soft diet, and the long chewing cycle. Interestingly, the teeth were structurally normal with no evidence of structural defects in the enamel or dentine. Our patient demonstrated generalized gingival enlargement; the cause for this enlargement is not known but it could be related to the presence of chronic gingival inflammation due to plaque accumulation. Our patient didn’t use anti-epileptic medications which might result in drug induced gingival overgrowth.

We previously reported the absence of lingual frenum in patients with Shashi-Penna syndrome, another rare neurodevelopmental syndrome caused by mutations in the *ASXL* gene [16]. Our patient with PTHS also demonstrated the absence of lingual frenum (Fig. 1), and interestingly patients with Rett syndrome also have absent lingual frenum (Hassona; unpublished data). It might therefore be possible that lack of lingual frenum is a common oral sign in patients with neurodevelopmental syndromes. Further studies are needed to confirm this observation. The absence of lingual frenum was reported as a useful diagnostic tool in patients with classical and hypermobile Ehlers-Danlos syndrome [17].

The presence of learning disability combined with hypotonia and muscle weakness can significantly impair oral health in patients with PTHS. In addition, patients with PTHS might suffer from seizures which necessitate the use of medications with potentially negative consequences on oral health including drug induced gingival overgrowth or drug induced hyposalivation [18]. 

Poor oral health in patients with PTHS might cause pain, infection, sialorrhea, bad breath, loss of ability to eat, and systemic inflammation leading to impaired overall quality of life [19]. In fact, patients with PTHS often suffer from constipation and gastrointestinal dysmotility, this might be contributed, at least partially, by the inadequate chewing and the tendency to eat soft food because of the prolonged chewing cycle [15, 20]. Oral health care professionals, therefore, should be part of the multidisciplinary care approach for patients with PTHS. Tailored oral hygiene instructions, diet assessment, parents’ education regarding the most suitable method of oral hygiene practice, and physiotherapy to improve chewing pattern are necessary measures in patients with PTHS to minimize the risk of dental caries and maintain adequate oral function.

Oral healthcare management in patients with PTHS might be challenging because of the associated comorbidities including seizures, delayed motor development, visual anomalies, severe intellectual disability, and limited or absent speech [9–13]. Communication with those patients might be challenging especially when visual anomalies, speech defect, and intellectual disability co-exist; in these situations, both verbal and non-verbal communications are not possible, and dental assessment and treatment might be needed under general anesthesia. We were unable to provide comprehensive dental care for our patient without the use of general anesthesia because of the lack of communication and the inability to cooperate in the dental clinic. General anesthesia in patients with PTHS might be complicated by the presence of seizures, the use of medications, the abnormal breathing rhythm, and the presence of gastroesophageal reflux [15]. General anesthesia therefore should only be performed in a hospital setting and following a thorough pre-operative assessment. Furthermore, patients with PTHS usually exhibit dilated pupils with sluggish response to light, instability of temperature, and decreased distal circulation. These findings should be observed during the pre-operative assessment and taken into consideration during the intra-operative monitoring and post-operative recovery after general anesthesia. Furthermore, polysomnography should be considered in individuals with PTHS to exclude obstructive sleep apnea. Importantly, Scoliosis has been reported in 18% of individuals with PTHS and can arise during puberty but also at younger ages [2, 15, 21]. The presence of skeletal abnormalities might complicate endotracheal intubation and necessitate an alternative positioning for intubation. Our patient had scoliosis which necessitated the intubation in the semi-setting position; general anesthesia therefore should be performed by an experienced anesthesiologist in a hospital setting.

## Conclusion

Oro-dental findings in PTHS include wide U-shaped palate, relative microdontia, gingival enlargement, dental caries, and absent lingual frenum. Provision of dental care often necessitates the use of general anesthesia because of the severe intellectual disability, gross motor delay, and communication difficulties. Dental care under general anesthesia might be complicated by the abnormal breathing rhythm, the gastroesophageal reflux, the abnormal pupil response to light, and the decreased distal circulation. Thorough pre-operative assessment, and proper intra-operative and post operative monitoring are necessary for the safe delivery of dental treatment.

Findings of the present report are limited by the fact that it was based on a single patient only. In addition, it was difficult to compare our findings with other reports on the same syndrome or similar syndromes due to the scarcity of studies specifically reporting oro-dental findings and oral health care management in these conditions. Collaborative efforts to report oro-dental findings in patients with rare syndromes using a uniform methodology are highly encouraged. In addition, clinical experience in provision of oral healthcare in affected patients should be shared across oral healthcare professionals involved in the management of patients with special health care needs.

## Data Availability

Data are available from the corresponding author on a reasonable request.

## Declarations

### Experimental protocols

No experimental protocols were applied in the present study. No approval was therefore needed.
